# Type I Spontaneous Coronary Artery Dissection in a 33-Year-Old Male With Clinically Suspected Myopericarditis

**DOI:** 10.7759/cureus.38570

**Published:** 2023-05-05

**Authors:** Cullen Nisson, Yeily Hernandez Mato, Nimisha Lingappa, James Abraham

**Affiliations:** 1 Internal Medicine, Health Corporation of America (HCA) Florida Brandon Hospital, Brandon, USA; 2 Family Medicine, Community Health of South Florida, Miami, USA; 3 Psychology, University of Florida, Gainesville, USA; 4 Biomedical Sciences, Barry University, Hollywood, USA; 5 Osteopathic Medicine, Nova Southeastern University Dr. Kiran C. Patel College of Osteopathic Medicine, Davie, USA

**Keywords:** spodick's sign, type ii diabetes, idiopathic pericarditis, st elevations, left anterior descending artery (lad), atypical spontaneous coronary artery dissection, left heart catheterization, intravascular ultrasound (ivus), acute myopericarditis, spontaneous coronary dissection

## Abstract

We report a 33-year-old male with uncontrolled type II diabetes, and tobacco and marijuana use who presented with chest pain after a night of binge drinking and vomiting. ECG changes were consistent with acute pericarditis. Troponin levels were found to be significantly elevated and rising. The patient was immediately treated with acetylsalicylic acid (ASA), morphine, nitroglycerin drip, and heparin drip. Echocardiogram showed preserved ejection fraction (EF) without effusion. Coronary angiography demonstrated a type I spontaneous coronary artery dissection (SCAD) of the mid-left anterior descending artery (LAD) without significant coronary artery disease. Diagnostic intravenous ultrasound (IVUS) confirmed a type I SCAD with penumbra and a minimal luminal area of 10 mm^2^ of the mid-LAD without significant luminal narrowing. Percutaneous intervention was performed with ultrasound-guided penumbra aspiration thrombectomy. Medical therapy was started with aspirin and ticagrelor, high-intensity statin, metoprolol tartrate, lisinopril, colchicine, and insulin. A biopsy or cardiac MRI was not performed due to the resolution of the patient's symptoms. We conclude that the development of a type I SCAD in this patient was multifactorial in nature, including clinically suspected acute myopericarditis, uncontrolled type II diabetes mellitus, and binge drinking associated with vomiting.

## Introduction

Spontaneous coronary artery dissection (SCAD) is defined as the separation of the intramural layers of the coronary artery wall by an intramural hematoma, with or without an intimal tear [1]. SCAD is uncommon, and >90% of cases occur in female patients between the ages of 47 and 53. Data on SCAD in males is limited due to its low prevalence in males [2]. Numerous case reports describe an association between SCAD and several inflammatory disorders including systemic lupus erythematosus, inflammatory bowel disease, celiac disease, and sarcoidosis [1]. Precipitating factors include emotional stress, physical stress (e.g., extreme Valsalva maneuver, retching, vomiting, coughing, or isometric exercise), use of stimulants, and hormonal triggers (e.g., pregnancy) [1,3]. Males with SCAD often cite a physical stressor [2]. Pathological reports from autopsy have demonstrated focal infiltration of inflammatory cells, particularly eosinophils, limited to the layers of the adventitia and periadventitial tissues of the dissected epicardial coronary arteries and are notably absent in the intima and media [1]. Here, we present a young male with cardiac risk factors who presented with myopericarditis and type I SCAD.

## Case presentation

**Figure 1:**
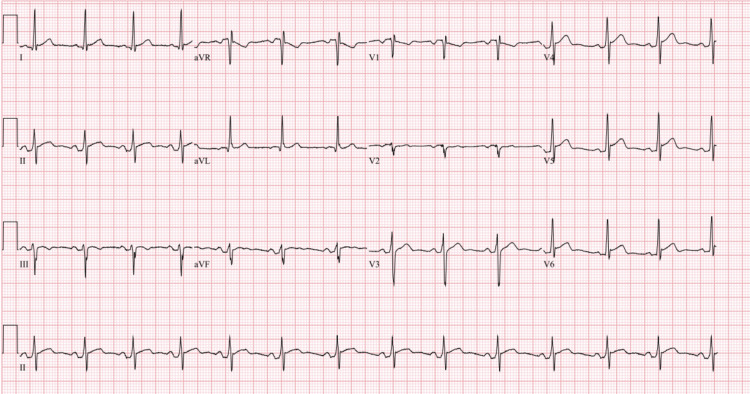
ECG upon presentation showed normal sinus rhythm with Spodick's sign (down-sloping TP segments), PR depressions, and ST segment elevations with normal concavity best seen in the inferolateral leads (I, II, III, aVF, and V3-V6). Reciprocal changes occurred in leads aVR and V1-V2. The ST segments were measured relative to the onset of the QRS complex because the TP segments were not isoelectric. ECG: electrocardiogram

**Figure 2:**
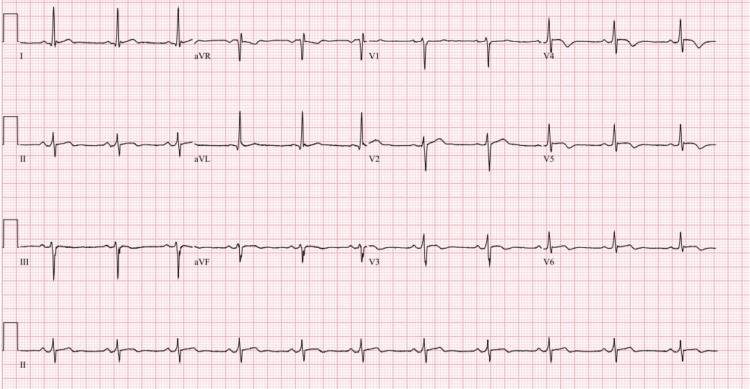
ECG nine hours later showed normalization of the TP, PR, and ST segments with diffuse flattened, biphasic, and inverted T waves consistent with resolving pericarditis. ECG: electrocardiogram

A 33-year-old African American male with uncontrolled type II diabetes mellitus diagnosed six months ago and nonadherent to metformin due to gastrointestinal (GI) adverse effects, seven-pack-year history of tobacco use, and marijuana use presented to the hospital for severe and burning substernal chest pain that began last night after binge drinking whiskey and two episodes of non-bloody, non-biliary vomiting. Initially, it was waxing and waning in nature and attributed to reflux, but this morning, it became constant and worse in severity. Vital signs included a heart rate of 83 bpm, blood pressure of 135/82 mmHg, respiratory rate of 18 breaths per minute, and 98% saturation on room air. The physical examination was unremarkable. On auscultation, S1 and S2 were present, and no murmurs, rubs, gallops, or friction rubs were appreciated. ECG upon arrival showed normal sinus rhythm with Spodick's sign (down-sloping TP segments), PR depressions, and diffuse ST elevations with reciprocal changes in leads aVR and V1, suggesting acute pericarditis (Figure 1). Troponins were 5,503 ng/L at the first hour, 8,284 ng/L at the second hour, 18,752 ng/L at the third hour, and peaked at 22,600 ng/L at the seventh hour. Other notable laboratory results included a hemoglobin A1c of 15.9% and a CRP of 1.34 mg/dL. All other laboratory results were normal (Table 1). He was immediately treated with 325 mg of acetylsalicylic acid (ASA), morphine, nitroglycerin drip, and heparin drip to empirically treat acute coronary syndrome. The patient's symptoms were almost completely resolved. An echocardiogram six hours later showed an ejection fraction of 60%-65% without wall motion abnormalities or effusion. ECG nine hours later showed normalization of the TP, PR, and ST segments with flattened, biphasic, and inverted T waves consistent with resolving pericarditis (Figure 2). CTA of the chest 10 hours later was negative for pulmonary embolism (PE), aortic dissection, and acute pulmonary process. Colchicine was started the next day to treat suspected myopericarditis.

**Table 1:** Laboratory studies. ACE: angiotensin-converting enzyme, ANA: antinuclear antibody, c-ANCA: centrally accentuated antineutrophil cytoplasmic antibody, CRP: C-reactive protein, ESR: erythrocyte sedimentation rate, HDL: high-density lipoprotein, LDL: low-density lipoprotein, TSH: thyroid-stimulating hormone

Laboratory studies	Values
Troponin I high sensitivity (normal range: <78 ng/L)	22,600 ng/L (at its peak), 409 ng/L (day 4)
Eosinophil count (normal range: 0.04-0.54 × 10^3^)	0 × 10^3^
Creatinine (normal range: 0.7-1.3 mg/dL)	0.95 mg/dL
Glucose (normal range: 70-110 mg/dL)	219 mg/dL
Hemoglobin A1c (normal range: <6.5%)	15.9%
Triglycerides (normal range: <150 mg/dL)	84 mg/dL
Total cholesterol (normal range: <200 mg/dL)	151 mg/dL
LDL (normal range: <100 mg/dL)	110 mg/dL
HDL (normal range: 40-60 mg/dL)	34 mg/dL
TSH (normal range: 0.36-3.74 uIU/mL)	1 uIU/mL
ESR (normal range: 0-15 mm/hour)	15 mm/hour
CRP (normal range: <0.30 mg/dL)	1.34 mg/dL
ACE (normal range: 14-82 U/L)	9 U/L
ANA pattern	Negative
Rheumatoid factor screen (normal range: <12)	<8.6 InU/mL
c-ANCA antibody (normal range: <0.9 AI)	<0.2 AI
Anti-myeloperoxidase (<0.9 AI)	<0.2 AI

**Figure 3:**
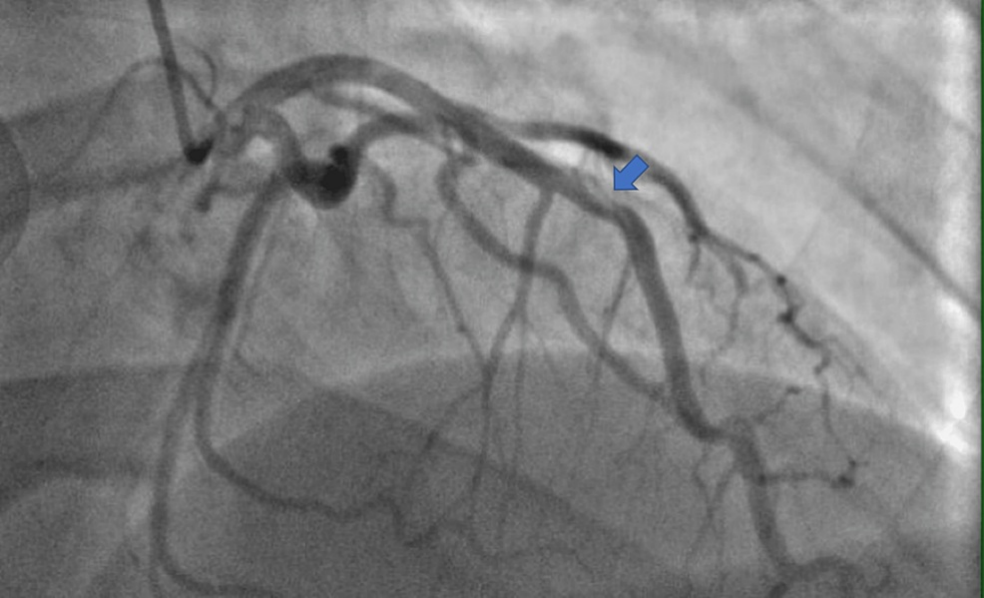
Right anterior oblique view demonstrating a type I SCAD of the LAD coronary artery (arrow). LAD: left anterior descending, SCAD: spontaneous coronary artery dissection

**Figure 4:**
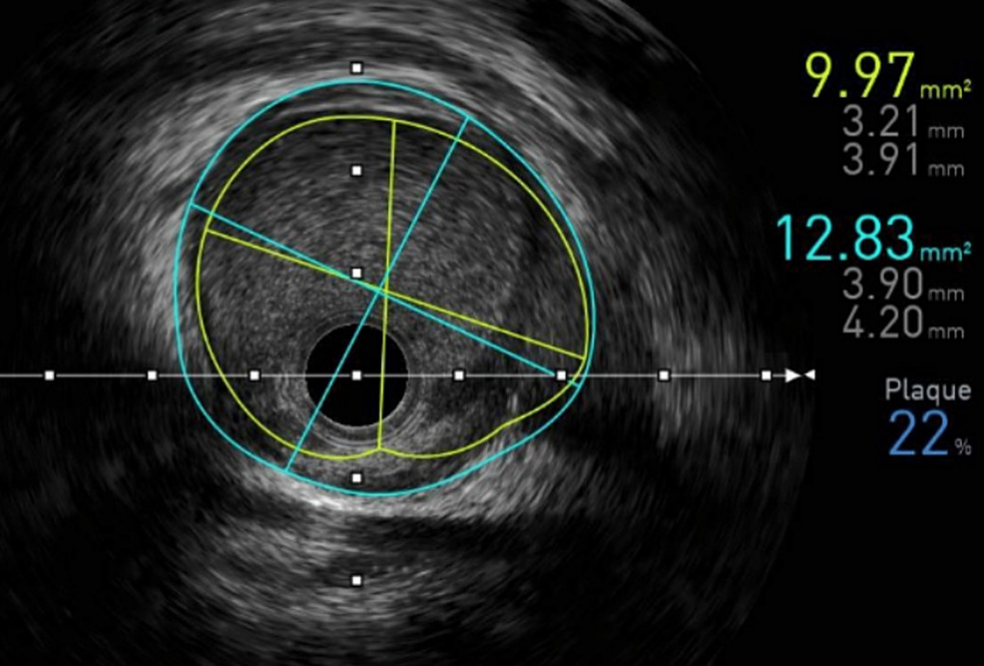
IVUS of the mid-LAD confirming a type I SCAD with a luminal area of 10 mm2. IVUS: intravenous ultrasound, LAD: left anterior descending artery, SCAD: spontaneous coronary artery dissection

Because this was a young patient with questionable acute coronary syndrome, we decided that the patient would benefit from a nonurgent left heart catheterization (LHC). If the LHC was normal, a cardiac MRI was decided to be the next step in the diagnostic workup. The risks and benefits were discussed with the patient, and we agreed to proceed with the procedure. LHC was performed 53 hours later with angiography showing a type I SCAD at the mid-left anterior descending artery (LAD) without significant luminal loss and 20% stenosis of the distal LAD. No significant coronary artery disease or tortuosity was noted (Figure 3). Diagnostic intravenous ultrasound (IVUS) was then performed and confirmed evidence of a type I SCAD with penumbra, a minimal luminal area of 10 mm^2^ of the mid-LAD without significant luminal narrowing, and a fibrofatty plaque distal to the lesion (Figure 4). Due to the patient's presentation of severe chest pain and significantly elevated troponin levels, an ultrasound-guided penumbra aspiration thrombectomy was performed with the removal of a maximum volume of 1 mL with one attempt and a maximum duration of five seconds. Post-thrombectomy angiogram or IVUS was not performed. Medical management was initiated with aspirin and ticagrelor, high-intensity statin, metoprolol, lisinopril, colchicine, and insulin. Further workup for connective tissue diseases and systemic inflammatory diseases was negative (Table 1). No biopsy or cardiac MRI was obtained due to the resolution of his symptoms, so we could not confirm a diagnosis of myocarditis. The patient was discharged. The patient did return four days later with recurring chest pain and anxiety with troponin levels of 409 ng/L at the time (Table 1) and was discharged without further workup.

## Discussion

SCAD is defined as the separation of the intimal layer away from the medial and adventitial layers of the coronary vessel wall. This has been noted to occur much more commonly in females than in males, with 90% of reported cases being female [1]. Data on SCAD in males is limited due to its low prevalence [2]. Some diseases associated with SCAD include fibromuscular dysplasia and several inflammatory disorders including lupus erythematosus, sarcoidosis, and inflammatory bowel disease [1]. However, the exact mechanism of dissection has not yet been clearly defined. It is proposed that prior focal infiltration of inflammatory cells into the epilayers of the vessel wall deteriorates the integrity of connective tissue and, when combined with prior damage to the intimal wall, creates the circumstances that allow a SCAD to develop [1-3]. Pathological autopsy reports in the literature have demonstrated focal infiltration of inflammatory cells, notably eosinophils, that were limited to the adventitia and periadventitial layers of the coronary artery with sparing of the intimal and medial layers. This pattern of focal infiltration of inflammatory cells differs from the pattern of involvement of intima and media seen in systemic arteritides such as polyarteritis nodosa or (eosinophilic) granulomatosis with polyangiitis [1].

Our patient had PR depressions, down-sloping TP segments, and non-focal ST elevations on ECG (Figure 1), an elevated CRP, significantly elevated troponin levels (Table 1) without focal wall motion abnormalities on ECHO, absence of coronary artery disease on angiography, and minimal loss of the lumen despite the presence of penumbra on IVUS (Figure 3). These findings were more suggestive of an acute myopericarditis process rather than an acute occlusive coronary artery syndrome [4]. The LAD in this patient likely had some intrinsic arteriopathy from uncontrolled diabetes and tobacco use. Using reported pathological autopsy reports in the literature, we propose that an episode of acute myopericarditis induced focal infiltration of inflammatory cells into the surrounding adventitia and periadventitial tissues of the epicardial coronary arteries predisposing the coronary artery to dissection [1]. The episode of binge drinking and vomiting likely created increased intrathoracic pressure and shearing forces that induced a SCAD. Workups for other commonly reported inflammatory diseases associated with SCAD were unrevealing (Table 1). Unfortunately, a biopsy or cardiac MRI was not obtained to confirm the etiology due to the resolving nature of the patient's condition. Placement of a stent was not performed due to complications reported in the literature [4].

## Conclusions

We present a case of this 33-year-old male with uncontrolled type II diabetes mellitus along with tobacco and marijuana use who developed a SCAD. It is still unclear in the literature what causes SCAD, particularly in males. We suspect that this patient developed myopericarditis, which played a significant role in the development of SCAD, although this diagnosis could not be confirmed without a biopsy or cardiac MRI. The cause of this patient's SCAD was likely multifactorial in nature. There are other factors that likely played a role, including uncontrolled type II diabetes mellitus, binge drinking and vomiting, and tobacco and marijuana use. We cannot conclusively say that myopericarditis was the cause of a SCAD to occur. We present this case in the hopes that if myopericarditis is identified as a cause of SCAD, early identification and treatment can prevent complications such as SCAD to occur. More studies would need to be done to explore the relationship between myopericarditis and SCAD.
